# Genotype–phenotype correlations in 9q34.3 microdeletion syndrome: a study of 35 Mainland Chinese patients

**DOI:** 10.1186/s13023-025-04076-6

**Published:** 2025-11-22

**Authors:** Tingting Yang, Danfeng Fang, Wenqi Zhu, Lili Wang, Weiqian Dai, Bing Xiao, Lixiao Shen, Yongkun Zhan, Yongguo Yu, Na Xu

**Affiliations:** 1 Department of Pediatric Endocrinology and Genetic Metabolism, Xinhua Hospital Affiliated to Shanghai Jiao Tong University School of Medicine, Kongjiang Road 1665, 200092 Shanghai, China; 2 Shanghai Institute for Pediatric Research, Shanghai, China; 3 Center of Clinical Genetics, Xinhua Hospital Affiliated to Shanghai Jiao Tong University School of Medicine, Shanghai, China; 4 Shanghai University of Medicine & Health Sciences, Shanghai, China; 5 MOE-Shanghai Key Laboratory for Children’s Environmental Health, Shanghai Jiao Tong University School of Medicine, Shanghai, China

**Keywords:** *EHMT1* gene, 9q34.3 microdeletion syndrome, Genotype-phenotype correlations

## Abstract

**Objectives:**

To provide the molecular characterizations and clinical profiles in patients with KLEFS1(Kleefstra syndrome 1) of Chinese ethic group and explore the genotype-phenotype correlation in this underrepresented population.

**Methods:**

A total of 35 Mainland Chinese patients with KLEFS1 were reported in the present study. The clinical data were assessed through reviewing medical records and standardized medical history questionnaires. We analyzed *EHMT1* variants and 9q34.3 microdeletion and performed genotype–phenotype correlation in two groups.

**Results:**

17 novel variants of *EHMT1* were identified, adding to the genetic landscape of the disorder. For the first time, we retrospectively describe the prenatal presentations and assess facial dysmorphisms in our cohort. There was no significant difference between the two groups in prevalence of clinical manifestations such as DD/ID, neurological symptoms, behavioral issues, obesity, congenital cardiac anomalies, male genital anomalies, or most other related clinical features, including developmental quotients (DQ). However, the frequencies of everted lower lip, small and spaced teeth, short neck, and renal anomalies were significantly higher among patients with deletions encompassing more than *EHMT1* compared to those with *EHMT1* variants (or deletions only disrupting *EHMT1*), with rates of 20% vs. 83.3% (*P* = 0.014), 14.3% vs. 80%(*P* = 0.017)13.3% vs. 66.7% (*P* = 0.031) and 5.3% vs. 42.9% (*P* = 0.047) respectively.

**Conclusions:**

This is the largest series of patients with KLEFS1 published to date in Mainland China. *EHMT1* haploinsufficiency contributes to the majority of important phenotypes of KLEFS1. Our findings enrich our knowledge of 9q34.3 microdeletion and enhance our comprehension of the pathogenic molecular mechanisms of *EHMT1*.

**Supplementary Information:**

The online version contains supplementary material available at 10.1186/s13023-025-04076-6.

## Introduction

Kleefstra syndrome 1(KLEFS1, [MIM: 610253]), also known as 9q34.3 microdeletion syndrome, is a rare genetic or chromosomal disorder characterized by a diverse range of clinical features [1]. Due to its complicated etiology, individuals with the syndrome present a broad spectrum of characteristic features, including neurodevelopmental disorders such as DD/ID, profoundly delayed speech, motor delay, attention deficit hyperactivity disorder (ADHD), autism spectrum disorder(ASD), epilepsy, and behavioral disorders, as well as various congenital anomalies like congenital heart defects, dysmorphic features, microcephaly, and renal anomalies [1, 2]. This syndrome is caused by deletions ranging from dozens of kilobases (kb) to about six megabases (Mb) in size in the 9q34.3 region [1, 3]. Nearly all identified cases involve deletions in the *EHMT1* (OMIM: 607001) gene, mapping to the distal end of 9q34.3. Furthermore, pathogenic variants of *EHMT1* have also been described [1, 4]. *EHMT1* encodes euchromatin histone methyltransferase 1, which plays a vital role in the Histone H3 lysine 9 (H3K9) methylation [5]. Currently, the hypothesis is that *EHMT1* haploinsufficiency is responsible for core phenotype of KLEFS1 [1, 4, 6]. However, this hypothesis cannot account for the wide clinical variability observed among KLEFS1 patients [7].

A few studies on genotype-phenotype correlation in KLEFS1 have been conducted, but their results are inconsistent [1, 4, 8–10]. None of the studies have comprehensively evaluated developmental quotient characteristics, including gross motor skills, fine motor skills, language, personal-social behaviors, and adaptive behaviors. Additionally, only ten KLEFS1 patients have been identified in Mainland China to date [11]. To address these gaps, we report on a cohort of 35 Mainland Chinese patients with KLEFS1, all of which have not been previously described. Our study aims to provide the molecular characterizations, clinical profiles (prenatal and postnatal), and conduct the genotype–phenotype analysis between patients with *EHMT1* variants (or deletions only disrupting *EHMT1*) and those with deletions encompassing more than *EHMT1* in this underrepresented population. We aimed to provide a more accurate estimated prevalence of KLEFS1 in China and to explore the contribution of *EHMT1* deficiency to the comprehensive phenotypes of 9q34.3 syndrome.

## Methods

### Cohort

Our research recruited 35 patients (or potential) diagnosed clinically and genetically with KLEFS1 through the China League of *EHMT1* Rare Disease in Mainland China from 2020 to 2024. Parents or guardians supplied genetic tests, reviewed medical records, and filled out a standardized medical history questionnaire. Information at prenatal and birth, the degree of developmental delay, MRI information, cardiovascular abnormalities, ophthalmological abnormalities, urogenital system abnormalities and gastrointestinal abnormalities were extracted from medical records. The questionnaire contained queries regarding the individuals’ stature, head circumference, developmental/neurological characteristics, irregular behaviors, and other clinical attributes. In addition, 21 families also submitted photographs showing the frontal and side views of their affected children’s facial features. To analyze the patients’ morphological characteristics, two clinicians independently evaluated their photographs.

### Developmental quotient assessment

The developmental quotient assessment was conducted in sixteen patients by evaluating their GDS scores. The schedules consisted of fine motor test, gross motor test, adaptive behavior test, language test and personal-social behavior test. The DQ scores was calculated on the average of the five scores.

### Genetic testing

Twenty-one patients with *EHMT1* mutations were identified through exome sequencing (ES) and subsequently validated by Sanger sequencing in all cases. Fourteen patients have deletions at 9q34.4 that include the *EHMT1* gene, identified using various examinations and platforms: CNV-seq (*n* = 2), SNP-array (*n* = 2) and ES (*n* = 10). Three deletions that solely affect *EHMT1* were confirmed by MLPA or QPCR (details in Table 1). Our research team conducted eight genetic tests, while the remaining tests were performed by outsourced laboratories.

**Table 1:** Summarization of *EHMT1* gene variants in 21 patients with KLEFS1

Patient	Variant location ^a^	Protein change	Type of variant	Inheritance	Literature report	ACMG classification	Additional genomic findings
P10	c.346 C >T (Het)	p. Gln116*	Nonsense	*De novo*		Pathogenic (PVS1 + PM6 + PM2_Supporting)	None
P11	c.673 C >T (Het)	p. Arg225*	Nonsense	*De novo*		Pathogenic (PVS1 + PM6 + PM2_Supporting)	None
P12	c.1468 C >T (Het)	p. Arg490*	Nonsense	*De novo*		Pathogenic (PVS1 + PM6 + PM2_Supporting)	None
P13	c.1468 C >T (Het)	p. Arg490*	Nonsense	*De novo*		Pathogenic (PVS1 + PM6 + PM2_Supporting)	None
P14	c.2107_2111del (Het)	p. Gly703Trpfs*59	Frameshift	*De novo*		Pathogenic (PVS1 + PM6 + PM2_Supporting)	None
P15	c.2041_2047dup (Het)	p. Asp683Glyfs*17	Frameshift	*De novo*		Pathogenic (PVS1 + PM6 + PM2_Supporting)	None
P16	c.2382 + 1G >T (Het)	/	Splice site	*De novo*		Pathogenic (PVS1 + PM6 + PM2_Supporting)	c.919–2 A >G(Hom)
							(*SLC26A4*)^b^
P17	c.2867 + 2T >C (Het)	/	Splice site	*De novo*		Pathogenic (PVS1 + PM6 + PM2_Supporting)	None
P18	c.2995del (Het)	p. Ala999Profs*11	Frameshift	*De novo*		Pathogenic (PVS1 + PM6 + PM2_Supporting)	None
P19	c.3036-1G >A (Het)	/	Splice site	*De novo*		Pathogenic (PVS1 + PM6 + PM2_Supporting)	None
P20	c.3036-6T >G (Het)	/	Splice site	*De novo*		Uncertain (PM6 + PM2_Supporting + PP3)	None
P21	c.3072del (Het)	p. Cys1025Valfs*22	Frameshift	*De novo*		Pathogenic (PVS1 + PM6 + PM2_Supporting)	None
P22	c.3072_3073del (Het)	p. Val1026Glnfs*150	Frameshift	*De novo*	#	Pathogenic (PVS1 + PM6 + PM2_Supporting)	None
P23	c.3310G >A (Het)	p. Glu1104Lys	Missense	Maternal(mosaic)	#	Likely Pathogenic (PM1 + PM6 + PM2_Supporting + PP3)	None
P24	c.3315_3316insA (Het)	p. Asn1106Lysfs*71	Frameshift	*De novo*	#	Pathogenic (PVS1 + PM6 + PM2_Supporting)	None
P25	c.3333 C >G (Het)	p. Cys1111Trp	Missense	*De novo*		Likely Pathogenic (PM1 + PM6 + PM2_Supporting + PP3)	None
P26	c.3589 C >T (Het)	p. Arg1197Trp	Missense	*De novo*	#	Likely Pathogenic (PM1 + PM6 + PM2_Supporting + PP3)	None
P27	c.3716 + 1G >C (Het)	/	Splice site	*De novo*		Pathogenic (PVS1 + PM6 + PM2_Supporting)	None
P28	c.1794dup	p. Asn599*	Nonsense	*De novo*		Pathogenic (PVS1 + PM6 + PM2_Supporting)	None
P31	c.1792–2 A >T	/	Splice site	Maternal(mosaic)		Likely Pathogenic (PVS1 + PM2_Supporting)	None
P33	c.3073T >C	p. Cys1025Arg	Missense	*De novo*		Uncertain (PM6 + PM2_Supporting + PP3)	None

^**#**^ mutation have been mentioned in previous report [1, 6, 12]. Het, Heterozygous. Hom, Homozygous

^a^ NM_024757.5

^b^ NM_000441.1

### Statistical analysis

Children were categorized into two groups to investigate the role of *EHMT1* haploinsufficiency. Group 1 consisted of individuals with *EHMT1* mutations or deletions only involving the *EHMT1* gene (22 patients). Group 2 included individuals with deletions encompassing more than the *EHMT1* gene (9 patients). Fisher’s exact two-sided test was used for the key phenotypes between the two groups; t-test was for Gesell Developmental Schedules (GDS) scores. Results were deemed statistically significant at *P* < 0.05 and of borderline significance at 0.05 < *P* < 0.10.

## Results

### Genetic analysis of 9q34.3 microdeletions and EHMT1 mutations

**Fig. 1:**
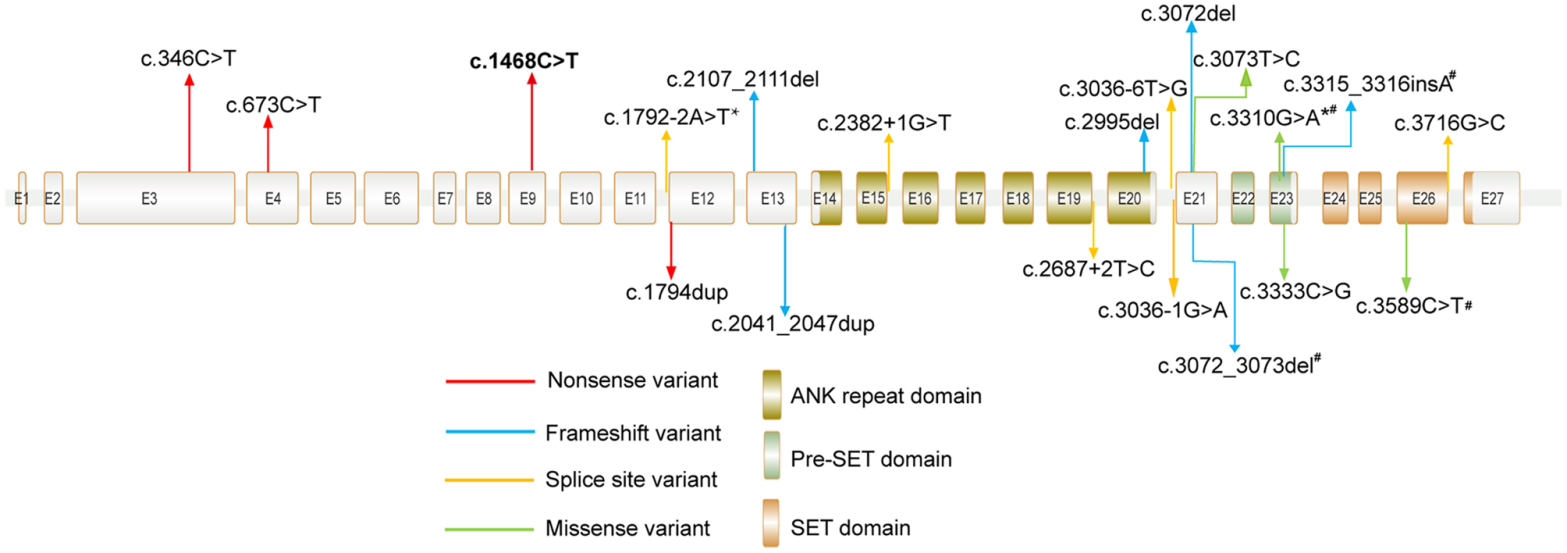
Distribution of *EHMT1* mutations in our cohort. Recurrent mutations are marked in bold. Protein domains are from UniPro. # mutations have been mentioned in previous report. *c.3310G > A variant in P23 and c.1792–2 A > T variant in P31 were inherited from their mothers who were in mosaic patterns

**Fig. 2:**
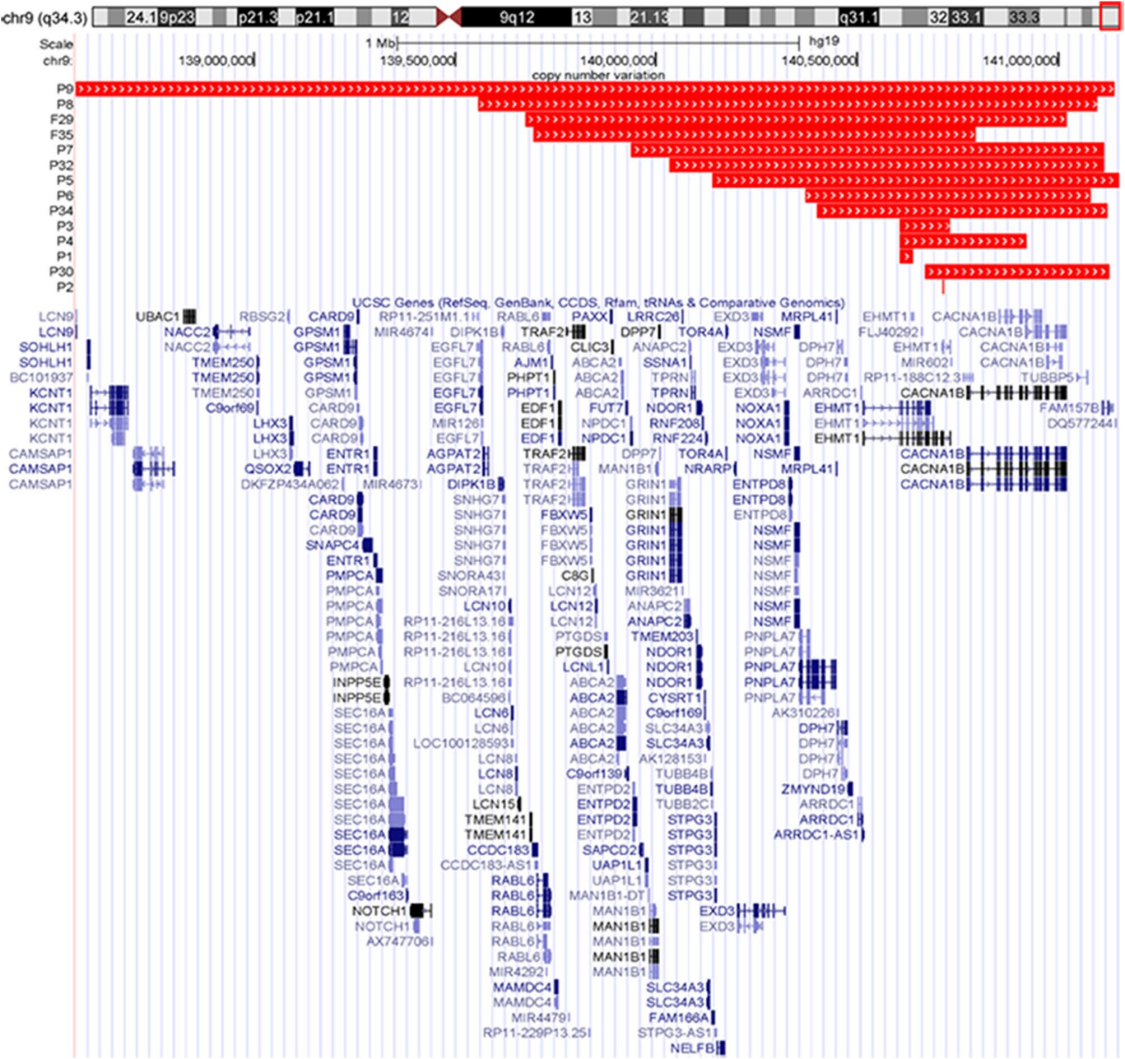
Distribution of deletions and duplications among 14 China mainland patients that varied from 0.12 kb to 2.58 Mb

Here we report 32 unrelated KLEFS1 patients and 3 fetuses diagnosed by different examinations. The 21 *EHMT1* point mutations of the present study are shown in Table 1 and Fig. 1. They comprise 5 nonsense mutations (two identical: c.1468 C > T), 6 frameshift mutations, 6 splice mutations and 4 missense mutations. Three of four missense mutants are located in pre- Su(var)3–9, Enhancer-of-zetste, trithorax (pre-SET) domain or SET domain, and one missense variant (c.3073T > C) occurred outside of these domains. 19 mutations are classified as pathogenic (P) or likely pathogenic (LP) and 2 were variants of uncertain significance (VUS) according to the American College of Medical Genetics and Genomics (ACMG) and Association for Molecular Pathology (AMP) criteria. Except for four variants (c.3072_3073del, c.3310G > A, c.3315_3316insA, c.3589 C > T), the remaining seventeen variants were unreported thus far. Besides, two subjects had additional genetic findings including *RET* and *SLC26A4*, P1 and P16 respectively. The size of 14 deletions is schematically displayed in Table 2 and Fig. 2. The largest size is 2.58 Mb overlapping many adjacent genes. The smallest one is 120 bp, exclusively covering exon 25 of the *EHMT1* gene. One fetus (F29) harbored terminal deletions accompanied by proximal duplications.

**Table 2:** Details of the 9q34.3 deletions in 14 individuals with KLEFS1

Patient	Ascertainment method	Rearrangement	Array coordinates (hg19)	Deletion or duplication size (kb)	Inheritance	Genes	Additional genomic findings
P1	MLPA	Deletion	140,605,391 − 140,638,567	33	NA	*EHMT1*	c.1858T > C(Het)
							(*RET*) ^a^
P2	Q-PCR	Deletion	140,712,491 − 140,712,611	0.12	NA	*EHMT1*	None
P3	MLPA	Deletion	140,605,244 − 140,729,148	124	NA	*EHMT1*	None
P4	WES	Deletion	140,605,375 − 14,0919,644	314	*De novo*	*EHMT1*,* CACNA1B*	None
P5	CNV-seq	Deletion	140,141,148 − 141,153,431	1012	*De novo*	Many	None
P6	CNV-seq	Deletion	140,370,935 − 141,080,050	709	NA	Many	None
P7	WES	Deletion	139,937,332 − 141,111,829	1174	*De novo*	Many	None
P8	WES	Deletion	139,559,141–141,093,906	1535	*De novo*	Many	None
P9	WES	Deletion	138,557,721 − 141,138,302	2581	*De novo*	Many	None
F29	SNP-array	Deletion and	139,674,181 − 141,018,648	1344	NA	Many	None
		Duplication	(525-3,512,648) ×3	3512			
P30	WES	Deletion	140,669,557 − 141,124,297	455	*De novo*	*EHMT1*,* CACNA1B*	None
P32	WES	Deletion	140,033,837 − 141,111,544	1077	NA	Many	None
P34	WES	Deletion	140,400,064–141,121,553	721	NA	Many	None
F35	SNP-array	Deletion	139,694,299 140,792,635	1098	*De novo*	Many	None

Het, Heterozygous. NA, not available. MLPA, Multiplex Ligation-dependent Probe Amplification. Q-PCR, Quantitative Real-Time PCR. CNV-seq, Copy Number Variation sequencing. WES, Whole Exome Sequencing. SNP, Single Nucleotide Polymorphism

a NM_020975

### Relatively high frequency of parental mosaic

**Fig. 3:**
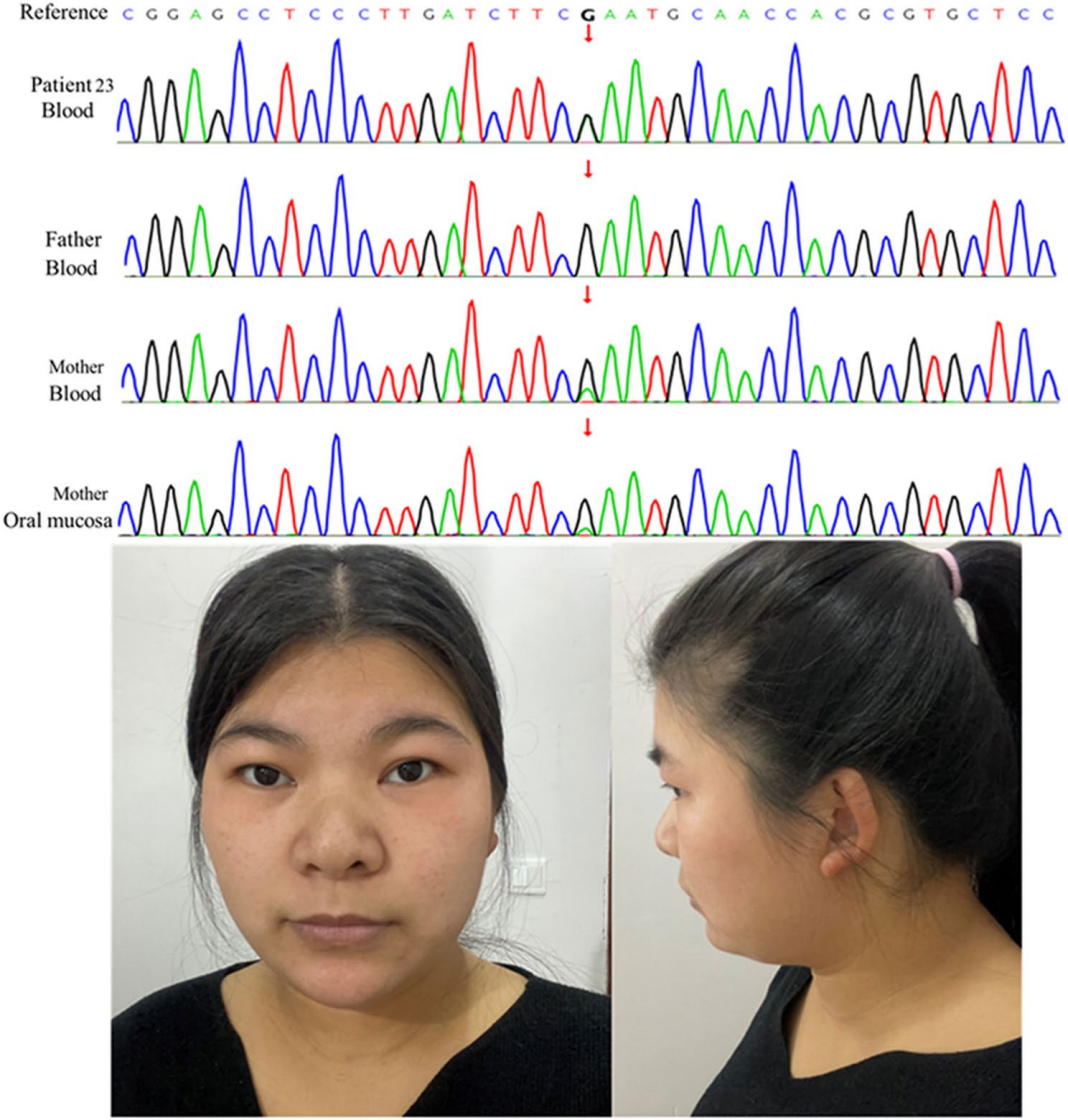
Familial segregation of *EHMT1* mutation c.3310G > A in Patient 23. The affected girl is heterozygous, her father is negative for this mutant, her mother has a mosaic pattern, as indicated by its reduced detection level in blood and oral mucosa using Sanger sequencing. The imaged of her mother shows no typical clinical presentation of KLEFS1 syndrome except for minor dysmorphic features and obesity. Her minor dysmorphic traits, including broad nasal bridge, thin upper lip and mild epicanthic fold

De novo occurrences of the mutations were verified by parental testing. Notably, the variant c.3310G > A (p. Glu1104Lys) in P23 was inherited from her mother with a mosaic pattern, as shown in Fig. 3. Her mother has no typical clinical presentation of KLEFS1 syndrome except for minor dysmorphic features and obesity. The c.1792–2 A > T mutation in P31 was inherited from her mother who is likely mosaicism (27%, Alt/Ref = 27/100), which was detected by trio-ES. In other words, two of the twenty-eight probands (7%, 2/28) were inherited from one of their parents who were the carriers of mosaic mutations in *EHMT1*. This is the first identification of a relatively high frequency mosaic carrier in parents of Chinese ethnicity who have reproduced proband with KLEFS1.

**Fig. 4:**
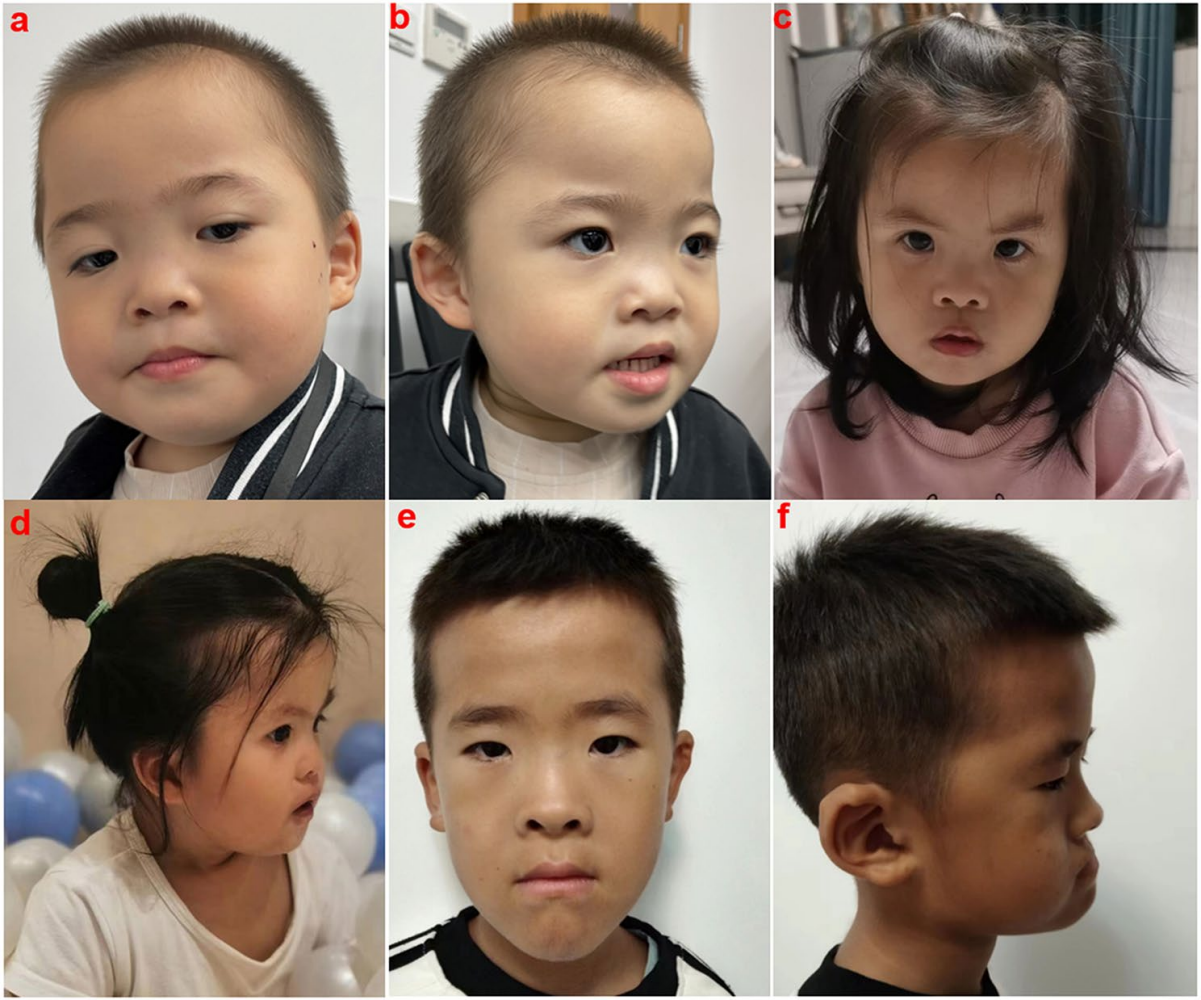
Dysmorphic images of three patients. Dysmorphic traits were listed as followings: (**a**) and (**b**) represented P4 who is harboring 9q34.3 deletion encompassing more than *EHMT1*(chr9: 140,605,375 − 14,0919,644). He has short neck, midface retrusion, synophrys, hypertelorism, long eyelashes, mild ptosis, depressed nasal bridge, short nose and everted lower lip. (**c**) and (**d**) represented P23 (c.3310G > A) and she has microcephaly, brachycephaly, midface retrusion, synophrys, hypertelorism, long eyelashes, epicanthic fold, depressed nasal bridge and everted upper lip with tented gesture. (**e**) and (**f**)represented P27 (c.3716 + 1G > C) and he has midface retrusion, synophrys, hypertelorism, epicanthic fold, broad nasal bridge and auricular anomalies

### Prenatal presentation

Total 26 cases’ clinical information regarding the prenatal period was available, covering three fetuses: F28, F29 and F35 (Table 3 and Supplementary Table S1). Among the KLEFS1 patients, normal ultrasound findings were present in 42.3% (11/26) of cases. Conversely, small for gestational age (SGA) (4/26, 15.4%) was the most frequent abnormality detected in prenatal ultrasound scans. Additionally, fetal brain abnormalities, small head circumference, polyhydramnios, and higher Systolic/Diastolic ratios (> 3.0) were also relatively common in KLEFS1 fetuses, each occurring in 11.5% (3/26) of cases. Other fetal structural abnormalities identified prenatally included cardiac defects (2/26, 7.7%), single umbilical artery (2/26, 7.7%), renal anomalies (1/26, 3.8%), congenital diaphragmatic hernia (1/26, 3.8%), and omphalocele (1/26, 3.8%).

**Table 3:** Prenatal feature of patients with KLEFS1 in our cohort

Feature	*N*	Total	%
Normal prenatal ultrasound	11	26	42.3%
Fetal brain abnormalities	3	26	11.5%
Cardiac defects	2	26	7.7%
Renal anomalies	1	26	3.8%
Decreased fetal movements	1	26	3.8%
Single umbilical artery	2	26	7.7%
Small for Gestational Age	4	26	15.4%
Small head circumference	3	26	11.5%
Omphalocele	1	26	3.8%
Polyhydramnios	3	26	11.5%
Congenital diaphragmatic hernia	1	26	3.8%
Short nasal bone	1	26	3.8%
Higher S/D ratios (> 3.0)	3	26	11.5%

S/D, Systolic/Diastolic

For F28, structural abnormalities were clearly detected in the 25th week of pregnancy, including anomalies in the fetal umbilical artery, umbilical hernia, and kidney (duplex kidney and pyelectasis on the right side). Moreover, abnormalities were observed in the brain, including thinning of the cerebral cortex and mild dilation of the lateral ventricles. These findings signify a severe condition of the fetal. For F29, abnormalities were identified during the 24-week sonographic assessment, including omphalocele and ventricular septal defect. These findings indicate developmental issues affecting both the abdominal wall and the heart. Moreover, F35 was found short nasal bone at 15 weeks of gestation(0.15 cm) and identified the 1098 kb deletion at 9q34.3. All the fetuses were terminated following genetic counseling and interpretation of Kleefstra syndrome.

### Overall prevalence of clinical manifestations of live patients

Due to termination for the three fetuses and the absence of clinical data for P21, they were excluded from subsequent statistical analyses. Accordingly, the primary analysis was conducted on 31 patients diagnosed with KLEFS1 (Supplementary Table S2 and S3). The most frequently observed characteristics among our cases (as detailed in Table 4) included DD/ID (31/31, 100%), speech delay (29/30, 96.7%), motor delay (29/30, 96.7%), specific behavioral problems such as stereotyped behaviors (12/26, 46.2%), and developmental/neurological issues such as MRI abnormalities (12/24, 50%), hypotonia (including neonatal or postnatal) (12/26, 46.2%) and chewing difficulties (10/23, 43.5%). In addition, delayed eruption of teeth (11/18, 61.1%), gait abnormalities (8/20, 40%), congenital heart defect (10/26, 38.5%) and constipation (9/24, 37.5%) were also common characteristics.

**Table 4:** Frequency of clinical features of present patients with KLEFS1 and frequency in literature

Feature	*N*	Total	%	Literature frequency ^a^ (%)
Neonatal problems				
Intrauterine Growth Restriction	5	26	19.2	
Premature	6	26	23.1	
Neonatal asphyxia	4	26	15.4	
Neonatal feeding difficulties	5	26	19.2	43
Neonatal hypotonia	5	26	19.2	42
Gastroesophageal reflux	3	26	11.5	23
Neonatal asphyxia	4	26	15.4	
Growth parameters				
Short stature	1	22	4.5	33^b^
Obesity	3	22	13.6	60^b^
Developmental/neurological				
DD/ID	31	31	100	91
Motor delay	29	30	96.7	
Speech(age ≥ 3)				
Absent speech	6	15	40	
Single words or phases	3	15	20	
Sentences	6	15	40	
Developmental regression	1	29	3.4	11^c^
Postnatal hypotonia	10	26	38.5	42
Hypertonia or hypotonia progressed to hypertonia	3	24	12.5	
Anomalies on brain MRI	12	22	54.5	59.6^d^
EEG abnormalities	2	24	8.3	
Seizures (including febrile seizures)	5	26	19.2	30^e^
Gait abnormalities	8	20	40.0	
Chewing difficulties	10	23	43.5	
Behavioral features				
Autism(age ≥ 3)	3	14	21.4	72
Hyperactivity	4	26	15.4	13
Stereotyped behavior	12	26	46.2	4.6
Sleep disturbance	3	26	11.5	
Drooling	7	26	26.9	
Self-injury	4	26	15.4	1.5
Crying for no cause	1	26	3.8	
Skin				
Eczema	2	26	7.7	
Cafe-au-lait spots	2	26	7.7	
Hypertrichosis	3	26	11.5	
Sparse hair	1	26	3.8	
Others				
Congenital heart defect	10	26	38.5	31
Hearing impairment	6	29	20.7	29
Recurrent infections	6	27	22.2	46
Ametropy	7	28	25.0	
Nystagmus	1	29	3.4	
Retinal lesions	1	31	3.2	
Delayed eruption of teeth	11	18	61.1	
Small and spaced teeth	6	19	31.6	
Constipation	9	25	36.0	47
Genital anomalies(male)	2	15	13.3	30^e^
Renal anomalies	5	28	17.9	10-30^e^
Scoliosis	2	26	7.7	11
Pes valgus	5	26	19.2	31
Flatfoot	4	26	15.4	
Congenital laryngomalacia	1	27	3.7	
Tracheomalacia	0	26	0	4
Umbilical/inguinal hernia	0	26	0	14.6
Anal atresia	0	26	0	3.4

a Frequencies based on Dmitrijs Rots, et al. [9]

b Frequencies based on Bouman A, et al. [13]

c Frequencies based on Zoë J. Frazier, et al. [10]

d Frequencies based on M.H. Willemsen, et al. [1]

e Frequencies based on the Gene Review [14]

### Neonatal features

In our group of patients with KLEFS1, the average gestation period was 38.7 ± 2.40 weeks, and six (6/26, 23%) patients experienced preterm birth. The newborn patients had an average weight of about 3.0 ± 0.77 kg and an average length of about 48.9 ± 4.09 cm. Five (5/26, 19%) patients had a history of intrauterine growth retardation. Additionally, six (6/26, 23%) patients had neonatal onset hypotonia and five (5/26, 19%) had feeding difficulties in newborn period. Newborn asphyxia was observed in four (4/26, 15.4%) patients and gastroesophageal reflux in three (3/26, 11.5%). Another symptom that occurred less often was post-term birth (P24) and hydrocephalus (P1).

### Developmental delay and Language impairment

DD/ID was assessed by reviewing the medical records or questionnaire in 31 patients and 21 of those were evaluated the degree of DD/ID. The vast majority of 31 cases experienced delayed motor development (29/30, 96.7%). The latest age at which patients achieved independent walking was 6 years old, despite the average age at which patients reached this milestone being 32 months. One patient (P3) had an extremely severe DD, while the majority of them (15/21, 71.4%) showed moderate to severe levels. Five cases (5/21, 23.8%) were diagnosed as mild DD.

Notably, one (1/29, 3.4%) exhibited development regression over 2 years old, particularly in language expression. Language impairment was evidently observed in all individuals. 40% of our cohort (6/15) had no speech at the investigation when they over the 3 years old, while 9 individuals were able to communicate using words, phrases, or simple sentences. The degree of language impairment varied from mild to severe based on language quotients of 18 patients, with ten of them presenting severe delay and five exhibiting moderate delay.

### Neurological and behavioral abnormalities

Among the series, 22 patients underwent brain MRI scans. In total, 12/22 (54.5%) patients displayed visible abnormalities on the MRI, and the most observed brain abnormalities were enlarged lateral ventricle (7/12, 58.3%) and demyelination (5/12, 41.7%). Periventricular leukomalacia and generous extracerebral space were each identified in three patients (3/12, 25%). Moreover, thinning of corpus callosum was detected in two patients (2/12, 16.7%). In addition, hypotonia (occurred in neonatal or postnatal) and poor chewing ability is another relatively common feature (12/26, 46.2% and 10/23, 43.5%). Five participants (5/26, 19.2%) had a history of seizures, but no epileptiform waves were detected on electroencephalogram (EEG), although two patients (2/24, 8.3%) had abnormal EEG findings. This may suggest mild or benign seizures, which typically carry a good prognosis. P9 had a history of febrile seizures at 5 months, while P10, P14, and P33 presented with primarily autonomic symptoms: P10 had eyelid blinking accompanied by involuntary twitching of the corner of the mouth at 1 year old, P14 had eyelid blinking at 5 months, and P33 had facial twitching at 5 months. No pharmaceutical intervention was administered to these children; instead, their condition resolved spontaneously after several months. However, P24 presented with more severe seizures characterized by upward eye deviation. Her electroencephalographic EEG tracing showed paroxysmal atypical spikes and/or spike-and-slow waves. She was prescribed levetiracetam to control her seizures at 2 years of age, and no drug resistance was observed. These non-severe conditions were in accordance with previously reported ones [15, 16]. At the time of evaluation, eight children (8/20, 40%) exhibited abnormal gait despite being able to walk independently, displaying toe-walking, out-toeing gait, or arm swaying while walking. Hypertonia was less frequent (3/24, 12.5%). Moreover, 46.2% of patients were reported to show stereotypic behavior which is common behavior issue among these patients. Other behavior problems include drooling (7/26, 26.9%), hyperactivity (4/26, 15.4%), autism (3/14, 21.4%), sleep disturbance (3/26, 11.5%), self-injury (3/26, 11.5%), unprovoked crying (1/26, 3.8%), easily startle (1/26, 3.8%).

### Other clinical features and first reported features

38.5% of patients (10/26) had congenital heart defect and the common gastrointestinal abnormality was constipation (9/24, 37.5%). Small and spaced teeth (6/19, 31.6%), ametropy (7/28, 25%), recurrent infections (6/27, 22.2%) and hearing impairment (6/29, 20.7%) were additionally frequent in our cohort. In addition, two boys were noted to have abnormality in the urogenital system- hydrocele(P4) and unilateral cryptorchidism(P22). Nystagmus and congenital laryngomalacia were unfamiliar, respectively in only one patient.

For the first time, we observed that several patients exhibited skin manifestations including hypertrichosis (3/26, 11.5%), cafe-au-lait spots (2/26, 7.7%), eczema (2/26, 7.7%), and sparse hair (1/26, 3.8%). The prevalent skeletal anomalies were pes valgus (6/26, 23.1%), which was barely observed in the published cases. Besides, delayed eruption of teeth (61.1%) is one of the most frequent characteristics that never been described before. Furthermore, P8 was the first reported with bilateral retinal lesions at birth, specifically tractional retinal detachment in the left eye and epiretinal membrane in the right eye. And no further pathogenic variants involving other genes or chromosomes were detected in these mentioned patients.

### Facial dysmorphisms

Dysmorphology examinations were conducted on 21 patients by two clinicians (Supplementary Table S4). This is the first detailed assessment of dysmorphic facial traits in KLEFS1 cohort. Except for P1, all individuals had distinctive facial features (20/21, 95.2%). As shown in Table 5, the predominant features observed were depressed nasal bridge (16/21, 76.2%), hypertelorism (15/21, 71.4%), midface retrusion (8/21, 38.1%), everted lower lip (8/21, 38.1%) and long eyelashes (7/21, 33.3%), followed by brachycephaly (6/21, 28.6%), short neck (6/21, 28.6%), long philtrum (5/21, 23.8%), synophrys (5/21, 23.8%), everted upper lip (6/21, 28.6%), epicanthic fold (4/21, 19%), microcephaly (4/21, 19%) and prominent forehead (4/21, 21%). Dysmorphic images of three patients were displayed in Fig. 4.

**Table 5:** Dysmorphic features in patients with KLEFS1 in our cohort

Dysmorphic characteristics	*N*	Total	%	Literature frequency (%)
Microcephaly (< 3rd percentile)	4	21	19.0	20^a^
Brachycephaly	6	21	28.6	
Short neck	6	21	28.6	
Midface retrusion (or mild)	8	21	38.1	
Prominent forehead	4	21	19.0	
Short philtrum	2	21	9.5	
Long philtrum	5	21	23.8	
Low-set ears	2	21	9.5	
Auricular anomalies	2	21	9.5	
Synophrys	5	21	23.8	
Arched eyebrows	2	21	9.5	
Hypertelorism	15	21	71.4	
Long eyelashes	7	21	33.3	
Upslanting palpebral fissures	1	21	4.8	
Downslanting palpebral fissures	1	21	4.8	
Ptosis	2	21	9.5	
Epicanthic fold	4	21	19.0	
Depressed nasal bridge	16	21	76.2	
Anteverted nares	6	21	28.6	
Broad nose (including Alae and Nasal Bridge)	4	21	19.0	
Short nose	4	21	19.0	
Everted upper lip	6	21	28.6	
Everted lower lip	8	21	38.1	
Carp-shaped mouth	1	21	4.8	
Macroglossia	1	21	4.8	

a Frequencies based on Dmitrijs Rots, et al. [9]

### Analysis on phenotype frequency between the two groups

To further elaborate on the role of *EHMT1* in 9q34.3 microdeletion syndrome, we compare the frequency of phenotypes between patients with variants or deletions affecting only *EHMT1* (Group 1) and those with deletions encompassing more than the *EHMT1* (Group 2). As depicted in Table 6, there was no notable difference among the two groups in the prevalence of DD/ID, neurological features, congenital cardiac anomalies, genital anomalies of males, renal anomalies, behavior issues, and other clinical features(*P* > 0.05). These results suggested the haploinsufficiency for *EHMT1* may contribute to the DD/ID, hypotonia and common medical comorbidities.

**Table 6:** Comparison of the prevalence of phenotypes between patients with disruptions of *EHMT1* alone and 9q34.3 deletions encompassing more than *EHMT1* gene

Clinical features	Disruptions of EHMT1 alone	9q34.3 deletions encompassing more than EHMT1 gene	*P* value ^a^
Developmental/neurological			
DD/ID	22/22(100.0%)	9/9(100.0%)	-
Motor delay	20/21(95.2%)	9/9(100.0%)	1
Speech delay	19/20(95.0%)	9/9(100.0%)	1
Hypotonia (neonatal or postnatal)	9/18(50.0%)	3/8(37.5%)	0.683
Developmental regression	1/21(4.8%)	0/8(0.0%)	1
Anomalies on brain MRI	7/16(43.8%)	4/6(66.7%)	0.635
EEG abnormalities	2/18(11.1%)	0/6(0.0%)	1
Seizures (including febrile seizures)	5/19(26.3%)	1/7(14.3%)	1
Gait abnormalities (walking independently)	6/15(40.0%)	2/5(40.0%)	1
Chewing difficulties	6/17(35.3%)	4/6(66.7%)	0.341
Behavioral features			
Autism(age ≥ 3)	3/16(18.8%)	0/3(0.0%)	1
Hyperactivity	3/18(16.7%)	1/7(14.3%)	1
Stereotyped behavior	9/19(47.4%)	4/7(57.1%)	1
Sleep disturbance	1/19(5.3%)	2/7(28.6%)	0.167
Drooling	5/19(26.3%)	2/7(28.6%)	1
Self-injury	3/19(15.8%)	1/7(14.3%)	1
Neonatal problems			
Intrauterine Growth Restriction	3/19(15.8%)	2/7(28.6%)	0.588
Premature	4/19(21.1%)	1/7(14.3%)	1
Neonatal feeding difficulties	2/19(10.5%)	3/7(42.9%)	0.101
Gastroesophageal reflux	1/19(5.3%)	2/6(33.3%)	0.133
Growth parameters			
Short stature	0/17(0.0%)	1/5(20.0%)	0.227
Obesity/Overweight	3/17(17.6%)	0/5(0.0%)	1
Dysmorphic features			
Microcephaly (< 3rd percentile)	2/15(13.3%)	2/6(33.3%)	0.544
Brachycephaly	5/15(33.3%)	1/6(16.7%)	0.623
Short neck	2/15(13.3%)	4/6(66.7%)	**0.031**
Depressed nasal bridge	11/15(73.3%)	5/6(83.3%)	1
Hypertelorism	11/15(73.3%)	4/6(66.7%)	1
Midface retrusion (or mild)	5/15(33.3%)	3/6(50.0%)	0.631
Everted lower lip	3/15(20.0%)	5/6(83.3%)	**0.014**
Everted upper lip	5/15(33.3%)	1/6(16.7%)	0.623
Long eyelashes	3/15(20.0%)	4/6(66.7%)	0.12
Anteverted nares	4/15(26.7%)	2/6(33.3%)	1
Long philtrum	4/15(26.7%)	0/6(0.0%)	0.281
Epicanthic fold	3/15(20.0%)	2/6(33.3%)	0.598
Synophrys	3/15(20.0%)	1/6(16.7%)	1
Prominent forehead	2/15(13.3%)	2/6(33.3%)	0.544
Short philtrum	2/15(13.3%)	0/6(0.0%)	1
Ptosis	0/15(0.0%)	2/6(33.3%)	0.071
Low-set ears	1/15(6.7%)	1/6(16.7%)	0.5
Auricular anomalies	2/15(13.3%)	1/6(16.7%)	1
Broad nose (including Alae and Nasal Bridge)	2/15(13.3%)	2/6(33.3%)	0.544
Short nose	1/15(6.7%)	3/6(50.0%)	0.053
Upslanting palpebral fissures	1/15(6.7%)	0/6(0.0%)	1
Downslanting palpebral fissures	0/15(0.0%)	1/6(16.7%)	0.286
Macroglossia	1/15(6.7%)	0/6(0.0%)	1
Carp-shaped mouth	1/15(6.7%)	0/6(0.0%)	1
Arched eyebrows	1/15(6.7%)	1/6(16.7%)	0.5
Other features			
Congenital heart defect	5/19(26.3%)	5/7(71.4%)	0.069
Hearing impairment	5/21(23.8%)	1/8(12.5%)	0.647
Ametropy	5/20(25.0%)	2/8(25.0%)	1
Delayed tooth eruption	7/14(50.0%)	4/4(100.0%)	0.119
Small and spaced teeth	2/14(14.3%)	4/5(80.0%)	**0.017**
Constipation	6/18(33.3%)	3/6(50.0%)	0.635
Recurrent infections	3/19(15.8%)	3/8(37.5%)	0.319
Genital anomalies(male)	1/10(10.0%)	1/4(25.0%)	0.505
Renal anomalies	1/19(5.3%)	3/7(42.9%)	**0.047**
Eczema	1/19(5.3%)	1/7(14.3%)	0.474
Cafe-au-lait spots	2/19(10.5%)	0/7(0.0%)	1
Hypertrichosis	2/19(10.5%)	1/7(14.3%)	1
Sparse hair	1/19(5.3%)	0/7(0.0%)	1
Scoliosis	0/19(0.0%)	2/7(28.6%)	0.065
Pes valgus	5/19(26.3%)	1/7(14.3%)	1
Flatfoot	3/19(15.8%)	1/7(14.3%)	1

a All the *P* values are Fisher’s Exact

However, the frequencies of everted lower lip, small and spaced teeth, short neck, and renal anomalies were significantly higher among Group 2 compared to those in group 1, with rates of 20% vs. 83.3% (*P* = 0.014), 14.3% vs. 80%(*P* = 0.017)13.3% vs. 66.7% (*P* = 0.031) and 5.3% vs. 42.9% (*P* = 0.047) respectively. The frequency of short nose (*P* = 0.053), scoliosis (*P* = 0.065), and congenital heart defect(*P* = 0.069) in the group 2 was slightly higher, approaching statistical significance at the borderline. These findings suggest that other genes may contribute to the multisystem effects observed in manifestations associated with 9q34.3.

### Analysis on DQ between the two groups

**Fig. 5:**
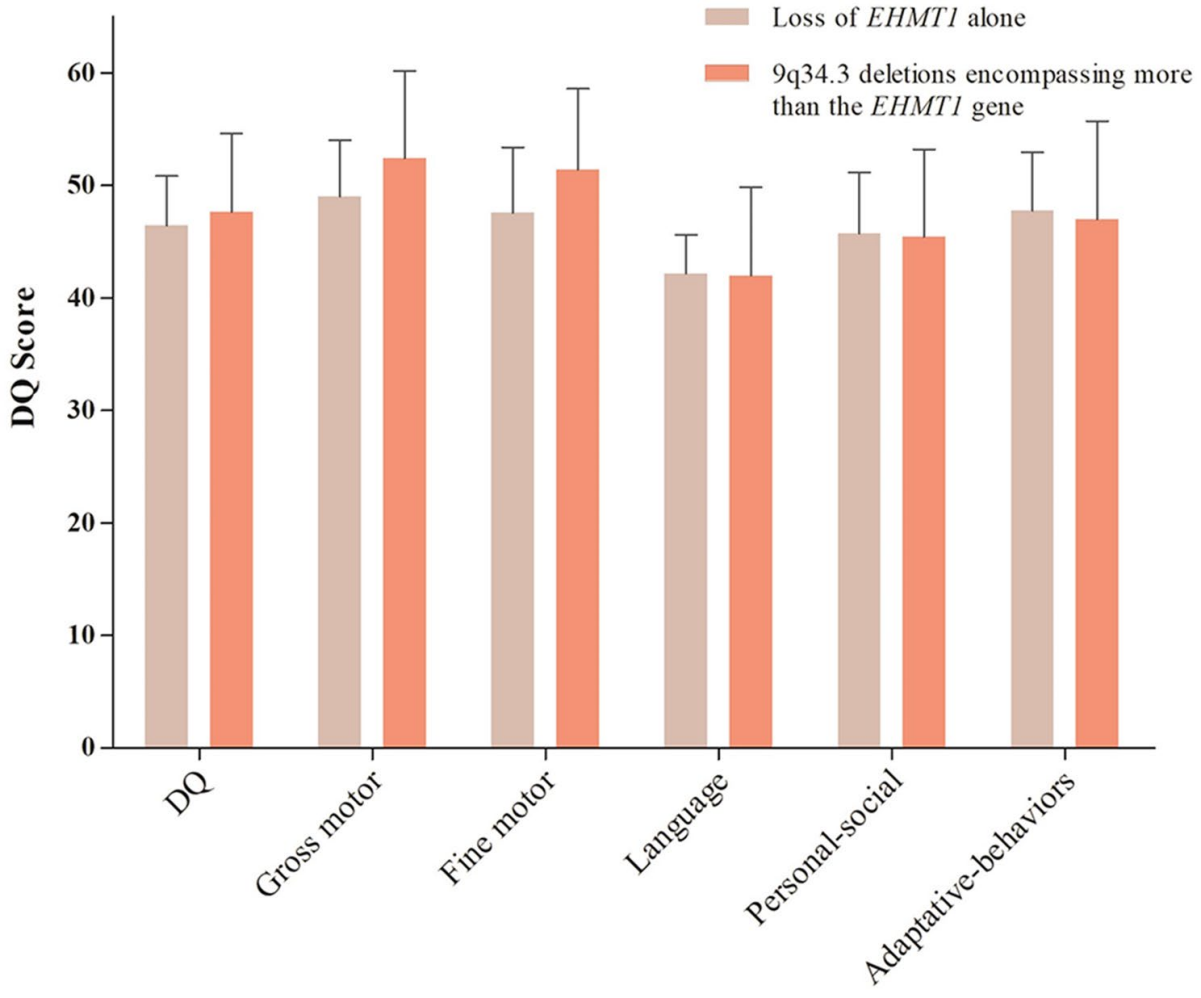
Gesell scores of DQ of 11 patients of our cohort. Group 1, patients with loss of *EHMT1* alone. Group 2, patients with 9q34.3 deletions encompassing more than *EHMT1* gene. DQ, developmental quotients

Furthermore, a comparative analysis of DQ was firstly performed between two groups in the current cohort. Sixteen patients, including 11 patients in Group 1 and 5 in Group 2, were assessed using the GDS, to investigate the impact of *EHMT1* on DQ. Poor speech ability was striking among them as the column of language quotient scores was short in Fig. 5.Overall, our findings showed no significant difference between the two groups in Gesell scores, specifically in the areas of gross motor skills, fine motor skills, language development, personal-social development, and adaptive behavior suggesting additional genes or regulatory regions proximal to *EHMT1* may not contribute to the severity of DD. Gesell scores of each patient were presented in Supplementary Table S5.

## Discussion

This study analyzed 32 newly identified KLEFS1 patients and 3 potentially affected fetuses, comprising the largest cohort in the Chinese ethnic population. Meanwhile, we investigated the genotype-phenotype associations with the syndrome.

Based on our genetic findings, approximately 70% of cases (24 out of 35) showed intragenic *EHMT1* variants rather than 9q34.3 microdeletions, and the predominance of single-nucleotide variants (SNVs) or small-scale sequence alterations in *EHMT1* is in agreement with prior reports [9, 10]. This predominance may be attributed to the growing utilization of ES in clinical diagnostic settings in recent years. Of these intragenic variants identified in our cases, 20 out of 24 (above 80%) are LOF (loss-of-function) mutations. These mutations are predicted to disrupt the open reading frame, leading to NMD through alternative splicing events or premature translation termination [17]. *EHMT1* was known as lysine specific methyltransferase. Besides transactivation domain in N-terminus and cysteine-rich domain, it still contains three highly conserved protein domains: ankyrin repeat (ANK) domain, pre-SET domain and SET domain [18]. ANK domain binds to H3K9me1 and H3K9me2 marks on methylated histone H3 tails [19]. Pre-SET domain is characteristic by structural zinc ion cluster and dimerization interface [18]. SET domain located in C-terminal is capable of binding S-adenosylmethionine (SAM) and substrate Lys residue, developing highly specific protein recognition and extremely effective catalysis [20]. Given that all the pathogenic missense variants documented in literature occur in these highly conserved functional domains, we hypothesized that the pre-SET domain and SET domain might serve as hotspot regions. Ayumi Yamada et al. demonstrated that missense mutations of *EHMT1* resulted in severe impairment of histone methyltransferase (HMT) activity and presented defective heterocomplex formation with G9a [21]. In other words, functional deterioration may occur contributing to insufficient biological activity and structurally compromised protein. This could provide rational interpretation that patients who harboring pathogenic missense mutants still showed core symptoms of *EHMT1*. As to c.3310G >A, this is the first reported detection of mosaicism for a missense mutation in *EHMT1*. The mild manifestations, limited to minor dysmorphic features and obesity, align with previous observations in mosaic carriers [22, 23]. This suggests that KLEFS1 does not manifest until the mosaic degree in the germline or somatic is sufficiently high [23]. Our investigation, for the first time, revealed that approximately 7% of the probands were associated with parental mosaicism, which may lead to a high recurrence risk. Anneke de Boer et al. indicated that carriers with *EHMT1* mosaicism are susceptible to psychiatric disorders even if they are seemingly unaffected [3]. Therefore, it is essential to take necessary measures to prevent the occurrence of psychiatric disorders for these carriers.

To establish a baseline understanding of the phenotypic variability associated with Kleefstra syndrome 1, we conducted a comprehensive evaluation of thirty-five previously unreported patients, including three affected fetuses. We began by retrospectively analyzing the prenatal phenotypes in the cohort. We found that only 42.3% of the sufferers had normal prenatal ultrasound outcomes, implying that more than 50% of the KLEFS1 cases might be accurately identified ahead of time if prenatal ES is carried out following the detection of fetal ultrasound anomalies, especially intrauterine SGA. This finding could enhance the understanding of prenatal phenotypes, thereby helping to increase awareness of KLEFS1 among obstetricians.

All live patients evaluated in our cohort exhibited DD/ID, a finding that appears to diverge from previously reported frequencies [9, 10]. This discrepancy may be attributed to the fact that patients with variants in the N-terminal region tend to present with milder phenotypes [9]. In our study, only one patient (P1) harbored a variant located in the N-terminal region. The individual experienced mild developmental problems and was attending primary school at the time of participation in our research. Arianne et al. noted that KLEFS1 is associated with childhood-onset overweight/obesity and endocrine-metabolic disorders [13]. However, in our mainland China cohort, the frequency of obesity was very low, and no endocrine-metabolic abnormalities were observed. This divergence may be attributed to ethnic differences. A similar pattern was observed for the low prevalence of short stature in our cohort, further supporting the potential role of ethnic background in shaping the phenotypic spectrum of KLEFS1-related traits. The frequencies of autism, stereotyped behavior, and self-injury were inconsistent with existing reporting, likely because the overwhelming majority of our patients were young children and behavior problems could be higher in the adult population. KLEFS1 patients exhibit increased vulnerability to severe psychiatric disorders [24, 25]. With this in mind, we propose monitoring or following up on their psychiatric symptoms or behavioral problems to ensure they receive optimal treatment.

Notably, speech disorders were a striking feature among our cohort, present in the absence of language or in the presence of only simple sentences—findings that align with previous research [26]. Moreover, anomalies on brain MRI, behavioral problems, dental issues, digestive and cardiac morbidity are common. Therefore, with the diagnosis of KLEFS1, it is essential to closely monitor all of the patient’s organ systems through a multidisciplinary team to detect potential multisystem complications. Although cardiac defects are common, no patients required cardiac surgery, suggesting that the cardiac defects are relatively mild or may gradually improve itself, with no instances of deterioration. As to Patient 17, her birth Apgar scores at 1, 5, and 10 min were 9, 9, and 9, respectively, because of hypotonia. However, at 5 months of age, she developed hypertonia. Two patients (P24 and P33) did not demonstrate hypotonia during the neonatal period but developed it as they grew older. Moreover, some patients developed hypotonia later, and others transition from hypotonia to hypertonia, highlighting the need for long-term follow-up and reassessment. This is a characteristic being reported for the first time. Previous observation pointed out that regression may occur in adulthood influenced by up-front sleep disturbance [27]. However, we noticed that one patient (Patient 2) manifested developmental regression in childhood, representing regression may come up during early life. Alongside the well-known phenotypes, our patients also presented nystagmus, retinopathy and congenital laryngomalacia. It would be valuable to investigate these phenotypes in more patients with *EHMT1* variants to determine if they are related to the syndrome or caused by an unidentified factor.

With the identification of an increasing number of patients in the Chinese population exhibiting smaller deletions and *EHMT1* variants, the genotype-phenotype analysis is necessary to compare the clinical features of patients harboring *EHMT1* mutations (or deletions solely affecting *EHMT1*) and those with deletions encompassing genes beyond *EHMT1*. Fisher’s exact two-sided test was utilized to evaluate the statistical significance of notable phenotypes between two cohorts, aiming to investigate genotype–phenotype associations. The analysis revealed no significant difference between the two groups in prevalent clinical manifestations such as DD/ID, neurological symptoms, behavioral issues, obesity, congenital cardiac anomalies, male genital anomalies, and the majority of other related clinical features, consistent with findings from previous studies [1, 4]. The data indicated that *EHMT1* haploinsufficiency influences these prevalent phenotypes.

Prior research suggested that patients with microdeletions exceeding 1 Mb exhibit a propensity for more severe ID [4]. But we demonstrated that there is no statistically significant difference in the severity of DD/ID by comparing GDS scores, a standardized developmental assessment. In other words, patients with deletions encompassing more than *EHMT1* don’t means more terrible DD/ID. This result highlights the pivotal role of *EHMT1* haploinsufficiency in the manifestation of DD/ID. Furthermore, our research determined that individuals with deletions encompassing genes beyond *EHMT1* are more likely to experience various facial dysmorphisms and renal anomalies, compared to those with *EHMT1* variants (or deletions only disrupting *EHMT1*). These findings highlight the contribution of additional gene(s) or region(s) of chromosome 9q34.3 beyond *EHMT1* within larger deletions to the severity and diversity of phenotypes. Future investigations should prioritize longitudinal studies to monitor the progression of these phenotypes and functional studies to elucidate the underlying biological mechanisms, particularly in the context of microcephaly and short stature.

There were several limitations to this analysis. Medical histories were obtained via medical record reviews and parental questionnaires, which may be susceptible to recall or information bias. Furthermore, standardized diagnostic assessments for ASD were not utilized, thus the analyses rely solely on medical record evaluations.

In summary, this largest series of KLEFS1 cases to data in Mainland China expand the genetic spectrum and detailed phenotypes of the syndrome, covering 17 novel mutations of *EHMT1* as well as some new manifestations. Moreover, *EHMT1* haploinsufficiency contributes to the majority of important phenotypes of KLEFS1, such as DD/ID, hypotonia, and most of other associated clinical features. Individuals with 9q34.3 microdeletion encompassing more than *EHMT1* and those with *EHMT1* variants (or deletions only disrupting *EHMT1*) may exhibit similar levels of DQ during childhood. However, patients with deletions encompassing more than *EHMT1* may present with additional anomalies, such as dysmorphic facial features and renal abnormalities. Our findings enrich our knowledge of 9q34.3 microdeletion and enhance our comprehension the pathogenic molecular mechanisms of *EHMT1*.

## Supplementary Information

Below is the link to the electronic supplementary material.

Supplementary Material 1

## Data Availability

All data are shown in this article.

## Abbreviations

KLEFS1: Kleefstra syndrome 1
EHMT1: Euchromatin histone methyltransferase 1
DD/ID: Developmental delay/intellectual disability
DQ: Developmental quotients
GDS: Gesell Developmental Schedules
EEG: Electroencephalogram
NMD: Nonsense mediated mRNA decay
ADHD: Attention deficit hyperactivity disorder
ASD: Autism spectrum disorder
H3K9: Histone H3 lysine 9
ES: Exome sequencing
MLPA: Multiplex Ligation-dependent Probe Amplification
Q-PCR: Quantitative Real-Time PCR
CNV-seq: Copy Number Variation sequencing
SNP: Single Nucleotide Polymorphism
VUS: Variants of uncertain significance
ACMG: American College of Medical Genetics and Genomics
AMP: Association for Molecular Pathology
LOF: Loss-of-function
ANK: Ankyrin repeat
SET: Su(var)3–9, Enhancer-of-zetste, trithorax
SAM: S-adenosylmethionine
HMT: Histone methyltransferase
